# Reciprocal Recurrent Genomic Selection Is Impacted by Genotype-by-Environment Interactions

**DOI:** 10.3389/fpls.2021.703419

**Published:** 2021-09-24

**Authors:** Maximilian Rembe, Jochen Christoph Reif, Erhard Ebmeyer, Patrick Thorwarth, Viktor Korzun, Johannes Schacht, Philipp H. G. Boeven, Pierrick Varenne, Ebrahim Kazman, Norman Philipp, Sonja Kollers, Nina Pfeiffer, C. Friedrich H. Longin, Niklas Hartwig, Mario Gils, Yusheng Zhao

**Affiliations:** ^1^Leibniz Institute of Plant Genetics and Crop Plant Research (IPK), Seeland, Germany; ^2^KWS LOCHOW GmbH, Bergen, Germany; ^3^KWS SAAT SE & Co. KGaA, Einbeck, Germany; ^4^Federal State Budgetary Institution of Science Federal Research Center “Kazan Scientific Center of Russian Academy of Sciences”, Kazan, Russia; ^5^Limagrain Europe, Ferme de l'Etang – BP3−77390, Verneuil-l'Ètang, France; ^6^Syngenta Seeds GmbH, Hadmersleben, Germany; ^7^State Plant Breeding Institute, University of Hohenheim, Stuttgart, Germany; ^8^Nordsaat Saatzucht GmbH, Langenstein, Germany

**Keywords:** grain yield, hybrid breeding, long-term selection gain, genotype-times-year interaction, abiotic stress

## Abstract

Reciprocal recurrent genomic selection is a breeding strategy aimed at improving the hybrid performance of two base populations. It promises to significantly advance hybrid breeding in wheat. Against this backdrop, the main objective of this study was to empirically investigate the potential and limitations of reciprocal recurrent genomic selection. Genome-wide predictive equations were developed using genomic and phenotypic data from a comprehensive population of 1,604 single crosses between 120 female and 15 male wheat lines. Twenty superior female lines were selected for initiation of the reciprocal recurrent genomic selection program. Focusing on the female pool, one cycle was performed with genomic selection steps at the F_2_ (60 out of 629 plants) and the F_5_ stage (49 out of 382 plants). Selection gain for grain yield was evaluated at six locations. Analyses of the phenotypic data showed pronounced genotype-by-environment interactions with two environments that formed an outgroup compared to the environments used for the genome-wide prediction equations. Removing these two environments for further analysis resulted in a selection gain of 1.0 dt ha^−1^ compared to the hybrids of the original 20 parental lines. This underscores the potential of reciprocal recurrent genomic selection to promote hybrid wheat breeding, but also highlights the need to develop robust genome-wide predictive equations.

## Introduction

Since the discovery of the advantages of hybrid breeding through increased performances due to the exploitation of heterosis (Shull, 1908), it has proven to be a successful strategy in allogamous species such as maize (Troyer, 1999), sunflower (Reif et al., 2013), sugar beet (Li et al., 2010), and rye (Geiger and Miedaner, 2015). Besides, hybrids display higher yield stabilities (Mühleisen et al., 2014), especially in marginal environments (Hallauer et al., 1988) and facilitate the stacking of major genes (Longin et al., 2012). These advantages stimulated investments in the implementation of hybrid breeding also in autogamous species, with the main challenge to develop economically competitive varieties that can compete against the line varieties on the market as the autogamous biology makes economic seed production challenging. Therefore, hybrid varieties must outperform significantly line varieties and the yield surplus must compensate for the higher costs in seed production. Recent advances enabled the introduction of hybrid breeding in autogamous species such as barley (Mühleisen et al., 2013), wheat (Melonek et al., 2021), and most successfully rice (Huang et al., 2017) but a major challenge is the selection gain per unit time: Classical hybrid breeding uses heterosis but exploits less additive variance and the breeding schemes are longer compared to line breeding (Longin et al., 2012).

A promising approach to breed high-yielding hybrids is to maximize the exploitation of beneficial heterosis. The concept of reciprocal recurrent selection (RRS) was originally proposed by Comstock et al. (1949) and optimizes the use of general and specific combining ability by selecting genotypes from one population based on the performance of their progeny resulting from crosses with another population. Ideally, this selection strategy results in a reciprocal shift in gene frequencies among the two populations from which female and male genotypes shall derive. Recurrent selection cycles are applied to further manifest this tendency. The success of RRS has been demonstrated in outcrossing species such as maize (Eyherabide and Hallauer, 1991; Tardin et al., 2007; Souza et al., 2010; Kolawole et al., 2018) and sugar beet (Doney and Theurer, 1978; Hecker, 1985). To the authors knowledge, no studies were published that investigate the potentials and limits of RRS in autogamous cereals such as wheat.

A disadvantage of RRS compared to recurrent selection is the elongation of breeding cycles due to the need to produce sufficient progeny based on which genotypes can be rated. In recurrent selection, the implementation of genomic selection has the potential to shorten the length of selection cycles and raise selection gain (Santantonio et al., 2020; Atanda et al., 2021), but empirical studies providing insights into the long-term effect in recurrent genomic selection are still missing. Research in animal breeding has suggested to complement RRS with genomic selection (Kinghorn et al., 2010). In oil palm, simulations have shown that genomic selection could potentially reduce the generation time of an RRS breeding cycle from 20 to 6 years (Cros et al., 2015). Integration of genomic selection into RRS would furthermore allow the combination of RRS and speed breeding approaches as proposed by Watson et al. (2018). Empirical evidence of the superiority of reciprocal recurrent genomic selection (RRGS) breeding programs, however, is still missing.

Many breeding programs are aimed at producing genotypes adapted to so-called mega-environments. Mega-environments are geographic regions that show similar growing conditions limiting the variance of the interaction effects between genotype and environments (Braun et al., 1996). In Germany, breeders generally aim for genotypes that are capable to meet the requirement criteria of the Federal Plant Variety Office (Bundessortenamt, Hannover), to release registered varieties. The Federal Plant Variety Office tests candidate genotypes in its official trials at up to 15 locations representing wheat growing regions in Germany. It is important to note here that Germany is not further subdivided in the Federal Plant Variety Office tests into target mega-environments for wheat breeding.

This study provides the first empirical results on the potential and limits of an RRGS breeding program in wheat targeted for Germany. The objectives were to (1) investigate the utility of genomic selection to identify superior females through genomic estimation of the general combining ability, (2) evaluate the selection gain for grain yield achieved by an RRGS breeding strategy, and (3) examine the impact of genotype-by-environment interaction on the effectiveness of a long-term breeding strategy.

## Materials and Methods

### Design of the Reciprocal Recurrent Genomic Selection Program

We implemented an RRGS program based on genomic and phenotypic data of a large hybrid wheat population (further denoted as HYWHEAT population) presented in detail in previous studies (Longin et al., 2013; Zhao et al., 2013, 2015; Gowda et al., 2014; Liu et al., 2016, 2020a,b; Jiang et al., 2017; Schulthess et al., 2018; Thorwarth et al., 2018, 2019). Briefly, 120 female and 15 male winter wheat lines adapted to Central Europe were crossed using chemical hybridization agents (e.g., Croisor 100; Kempe et al., 2014) applying standard in house protocols. 1,604 single-cross hybrids were produced. The 1,604 hybrids, their 135 parents, and 10 commercial varieties (As de Coeur, Colonia, Genius, Hystar, JB Asano, Julius, Kredo, Tabasco, Tobak, Tuerkis) were evaluated for grain yield in 11 environments, i.e., 5 and 6 locations (Adenstedt, Boehnshausen, Hadmersleben, Harzhof, Hohenheim, and Seligenstadt), in the growing seasons 2011/2012 and 2012/2013, respectively, in Central Europe, resulting in high quality phenotypic data (Supplementary Table 2 in Zhao et al., 2015). The 135 parental lines were genotyped using a 90,000 SNP array based on an Illumina Infinium assay and after quality tests, 17,372 high-quality SNP markers were retained. The phenotypic and the genomic data were combined, and a ridge regression best linear unbiased prediction (RRBLUP) model was trained fitting additive and dominance effects using the package rrBLUP (Endelman, 2011) in the R software environment (R Core Team, 2020). The implementation of the RRBLUP model was described in detail elsewhere (Zhao et al., 2015). Briefly, the model was:


(1) $$\begin{array}{r} Y=1_{n}\mu +Z_{A}a+Z_{D}d+e, \end{array}$$

where *Y* refers to the grain yield data of the 135 parent lines and their 1,604 hybrids, μ was the overall mean, 1_*n*_ was an *n*-dimensional vector of ones, *a* and *Z*_*A*_ denoted the additive effects and the corresponding design matrix, and *d* and *Z*_*D*_ denoted the dominance effects and the corresponding design matrix. The estimated *a* and *d* effects were used to predict the genotypic values of the hybrid performances when crossed with the 15 male lines.

In the recurrent genomic selection program, we focused on the female pool and selected 20 out of the 120 female lines. The selection was based on the first-year estimates of general combining abilities and further criteria such as for example being carrier of the dwarfing gene *Rht2*. The 20 female lines formed the C_0_ cycle and were crossed following a single round robin design (A x B, B x C, C x D, …, T x A), i.e., every line was used in two crosses resulting in 20 F_1_'s. The 20 F_1_'s were grown in the following season and selfed to the F_2_ generation in the green house. Seeds were harvested and around 30 F_2_ plants were grown for each of the 20 biparental families amounting to a total of 629 F_2_ plants. The 629 F_2_ plants were genotyped before flowering using the above-mentioned SNP array. The general combining abilities of the 629 F_2_ plants when crossed with the 15 original male lines were estimated using the SNP profiles and the above outlined RRBLUP model. The best 3 F_2_ plants per family, i.e., 60 F_2_ plants in total, were selected and selfed toward the F_5_ generation resulting in 2,886 F_5_ genotypes. Descendants from each of the 20 initial crosses were represented in this panel with a mean number of genotypes of 144, ranging from 76 to 277. Seeds of the 2,886 F_5_ genotypes were grown in single row plots in the season 2016/2017 and a fraction of 382 F_5:6_ families were visually selected based on overall agronomic performance (disease resistance) and considering plant height and flowering time to facilitate hybrid seed production when crossed with three out of the 15 above outlined male lines. The 382 F_5:6_ families were genotyped using the above-mentioned SNP array. The general combining abilities of the 382 F_5:6_ families when crossed with the 15 original male lines were estimated using the SNP profiles and the above outlined RRBLUP model. Based on the estimated general combining ability effects, 50 outstanding F_5:6_ families were selected (denoted as C_1_S). All of the 20 biparental F_2_ families were represented in this set of families.

As further reference point besides C_0_, 60 F_2_ plants out of the above outlined 629 F_2_ plants of the 20 biparental families were randomly selected. Here, a total of 3 F_2_ plants were randomly drawn from each of the 20 biparental families and selfed toward the F_5_ generation resulting in 714 F_5_ genotypes. Seeds of the 714 F_5_ genotypes were multiplied in single row plots in the season 2016/2017. A subfraction of 30 F_5:6_ families were visually selected considering plant height and flowering time to facilitate hybrid seed production when crossed with three out of the above outlined 15 male lines. The subfraction of 30 F_5:6_ families were denoted as C_1_R. The 30 genotypes of the C_1_R cycle were genotyped using the above-mentioned SNP array. The integrated data set was filtered by excluding markers with more than 5% missing values, resulting in 4,031 unique and polymorphic markers.

### Evaluation of the Selection Gain in Field Trials and Phenotypic Data Analyses

The data set comprised 376 genotypes, including 3 male lines previously used to produce the 1,604 original F_1_ hybrids, 20 female lines from C_0_, 49 female lines (one out of the above mentioned 50 lines were discarded because hybrid seed production failed entirely) from C_1_S, 30 female lines from C_1_R, 267 F_1_ hybrids, and 7 commercial varieties (Julius, Colonia, Tobak, Elixer, RGT Reform, Hystar, and Genius). The hybrids were derived by crossing the 99 female and the 3 male lines using a factorial mating design. For 267 of the potential 297 single-cross hybrids, enough seeds were harvested for intensive field trials.

All 376 genotypes were evaluated in yield plots for grain yield and plant height at 6 locations in the growing season 2018/2019. The locations were Hadmersleben (latitude 51.98 N, longitude 11.30 E), Mintraching (latitude 48.95 N, longitude 12.25 E), Adenstedt (latitude 52.20 N, longitude 10.18 E), Sossmar (latitude 52.2 N, longitude 10.08 E), Wohlde (latitude 52.8 N, longitude 9.98 E), and Boehnshausen (latitude 51.85 N, longitude 10.95) (Supplementary Table 1). The same seeding rate of 230 grains per m^2^ was used for both parental lines and hybrids. The plot size ranged from 7.2 to 12 m^2^. Harvesting was performed mechanically and adjusted to a moisture concentration of 140 g H_2_O kg^−1^. The field design was an alpha lattice with block size 11 where each environment corresponded to one replication. The yield trials were treated with fertilizers, fungicides, and herbicides according to farmers practice for intensive wheat production.

The quality of the outlier-controlled phenotypic data from each environment was assessed by estimating the genomic repeatability employing the package BGLR (Perez and de los Campos, 2014) in the software environment R (R Core Team, 2020). For this purpose, the following genomic prediction model was used for lines:


(2) $$\begin{array}{r} y=1_{n}\mu +g+e, \end{array}$$

where *y* was the *n*-dimensional vector of phenotypic records of each environment, 1_*n*_ was an *n*-dimensional vector of ones, *u* was a common intercept, *g* was an *n*-dimensional vector of additive genotypic values and *e* was the residual term. It was assumed that *u* was a fixed parameter, $g\sim N\left(0,G\sigma_{g}^{2}\right)$ and $e\sim N\left(0,I_{n}\sigma_{e}^{2}\right)$, where *I*_*n*_ denoted the *n*×*n* identity matrix and *G* denoted the *n*×*n* genomic relationship matrix among genotypes as proposed by VanRaden (2008). For each environment, a 5-fold cross-validation scheme was implemented. Therefore, the population of tested lines was randomly divided into five subsets of equal size. One subset was predicted after the model was trained based on the phenotypic and genotypic data from the remaining four subsets. The correlation between the observed and predicted values defined the prediction ability. After performing 100 5-fold cross-validations, genomic repeatability was obtained by the mean of the prediction abilities.

For assessing the quality of the outlier-controlled phenotypic data for the hybrids tested in each environment, genomic repeatability was estimated employing the following model using the package BGLR (Perez and de los Campos, 2014) in the software environment R (R Core Team, 2020):


(3) $$\begin{array}{r} y=1_{n}\mu +Z_{A}a+Z_{D}d+e, \end{array}$$

where *y* was the *n*-dimensional vector of phenotypic records of each environment, 1_*n*_was an *n*-dimensional vector of ones, μ was the common intercept, *a* and *Z*_*A*_ denoted the additive effects and the corresponding design matrix, and *d* and *Z*_*D*_ denoted the dominance effects and the corresponding design matrix. The cross validation of hybrids was executed in the same manner as described for lines.

After outlier tests, the following model was used to obtain best linear unbiased estimations (BLUEs) across environments:


(4) $$\begin{array}{r} y_{ijk}=\mu +\;g_{i}+r_{j}+b_{k}+e_{ijk}, \end{array}$$

where *y*_*ijk*_ referred to the phenotypic performance of the *ith* genotype at the *jth* location in the *kth* block, μ referred to the intercept, *g*_*i*_ referred to the genetic effect of the *ith* genotype, *r*_*j*_ referred to the effect of the *jth* location, *b*_*k*_ referred to the *kth* block in the *jth* location and *e*_*ijk*_ denoted the residual. Genotype was treated as fixed and the remaining effects as random. Outlier detection test was performed following the method M4r as described by Bernal-Vasquez et al. (2016), where the standardized residuals were used in combination with the Bonferroni-Holm test to identify an outlier. The detected outliers (3 for grain yield) were removed for further analysis. Moreover, we estimated variance components with the following model:


(5) $$\begin{array}{l} y_{imfnk}=\mu +a+l_{n}+b_{nk}+p_{i}+{g^{\prime }}_{f}+{g^{\prime \prime }}_{m}+g_{fm}\\ +{\left(g^{\prime }l\right)}_{fn}+{\left(g^{\prime \prime }l\right)}_{mn}+{\left(pl\right)}_{in}+e_{mfink}, \end{array}$$

where *y*_*ifmnk*_ referred to the phenotypic performance of the *ith* genotype at the *nth* location in the *kth* block, *l*_*n*_ referred to the *nth* location, *b*_*nk*_ referred to the *kth* block at the *nth* location, *p*_*i*_ referred to the effect of the *ith* parental line, ${g^{\prime }}_{f}$ referred to the general combining ability (GCA) effect of the *fth* female line, ${g^{\prime \prime }}_{m}$ referred of the GCA effect of the *mth* male line, *g*_*fm*_ referred to the specific combining ability (SCA) effect of the *fmth* genotype, ${\left(g^{\prime }l\right)}_{fn}$ referred to the interaction effect between the GCA of the *fth* female and the *nth* environment, ${\left(g^{\prime \prime }l\right)}_{mn}$ referred to the interaction effect between the GCA of the *mth* male and the *nth* environment, (*pl*)_*in*_ referred to the interaction effect of the *ith* parental line and the *nth* environment *e*_*mfink*_ referred to the residual. Dummy variables were used to distinguish between checks, lines, and hybrids. Based on the variance components, heritability (*h*^2^) was estimated separately for lines and hybrids as $h^{2}\;=\frac{\sigma_{G}^{2}}{\sigma_{G}^{2}+\frac{\sigma_{GxE}^{2}+\sigma_{e}^{2}}{l}}$, where $\sigma_{G}^{2}$ refers to the genetic variance of lines or hybrids, $\sigma_{GxE}^{2}$ refers to the genotype-by-environment variance $\sigma_{e}^{2}$ refers to the residual variance, and *l* denotes the average number of environments in which the genotypes were tested. Linear mixed models have been executed using ASReml version 4.0 (Butler et al., 2017) in the software environment R (R Core Team, 2020).

GCA_Female_-by-environment interaction effects were estimated by using the same model as in Equation (5) to further characterize the environments in which the genotypes were evaluated. The GCA_Female_-by-environment interaction effects were estimated for the experiments of the growing season 2018/2019 only and furthermore in a combined data set consisting of the training environments of the growing seasons 2011/2012 and 2012/2013 and the test environments of the growing season 2018/2019. The GCA_Female_-by-environment interaction effects were used to perform principal component analyses (PCA) and obtain Euclidean distances based on which the environments were clustered in a complete-linkage approach.

The observed response to selection was estimated as *R*_*obs*_ = Ŝ, where Ŝ = μ_*sel*_−μ_*pop*_ denoted the observed selection differential, with μ_*sel*_ being the phenotypic mean of the selected genotypes and μ_*pop*_ being the mean of the population from which the selected genotypes were drawn. The C_1_ hybrids of the underlying RRGS breeding program have been produced using female lines deriving from a population of 629 genotypes. The capacity for all of the 629 genotypes to produce hybrids has not been estimated in field experiments but only through genomic prediction. For this reason, the mean performance of the C_0_ hybrids evaluated in the growing season 2018/2019 has been considered as an approximation for μ_*pop*_.

The expected response to selection was estimated as *R*_exp_ = *i* • *h* • σ_*A*_, where *i* denoted the intensity of selection, *h* refers to the square root of the heritability, and σ_*A*_ denoted the standard deviation of the breeding values. Selection intensity was calculated as $i\left(N,G\right)=i\left(\alpha \right)-\frac{G-N}{2N\left(G+1\right)i\left(\alpha \right)}$, where *N* was the number of selected genotypes, *G* was the size of the population from which the selected genotypes were drawn, and $i\left(\alpha \right)=i\left(\frac{N}{G}\right)$ referred to the standardized selection differential according to tabulated values (e.g., Becker, 1975).

Selection was performed in two steps. In the first step, 60 F_2_ plants were selected out of a population of 629, resulting in a selection intensity of *i*(*N, G*) = *i*(60, 629) = 1.78. Since the selection was based on genomic predictions of the GCA effects of the female lines evaluated in the HYWHEAT experiments, the relevant variance of breeding values corresponds to $\sigma_{GCA}^{2}\;$, estimated in the experiments of the growing seasons 2011/2012 and 2012/2013 (Zhao et al., 2015). The selection was performed in a population of F_2_ plants derived from crosses of genotypes from the aforementioned population. Specifically, three F_2_ plants were selected from each family. From quantitative genetic theory, it can be inferred that half of the genetic variance can be exploited if a selection is performed within an F_2_ family (Hallauer et al., 2010). It follows that for the first step of selection, $\sigma_{GCA\text{\_}F2}=\sqrt{\frac{1}{2}\sigma_{GCA}^{2}}=1.2$. The square root of the heritability, *h*, was assessed using as a conservative estimate the prediction abilities obtained in a chessboard-like cross-validation considering two out of the three different test sets T_2_, T_1_, and T_0_: T_2_ test sets included hybrids sharing both parental lines, T_1_ test sets comprised hybrids sharing one parental line, and T_0_ test sets contained hybrids having no parental line in common with the hybrids in the related training sets. In the RRGS program, male testers were not changed and thus, the C_1_ lines reflected a mix between the T_1_ and T_2_ scenario with a prediction ability of 0.55 and 0.76, respectively. For simplicity, the mean of the prediction abilities for scenarios T_1_ and T_2_ was considered, resulting in *h* = 0.66.

In the second step of selection, 50 plants were selected from a population of 382 F_5:6_ plants. While *h* is considered equal to the first step, *i*(*N, G*) and σ_*GCA*_ changed, with *i*(50, 382) = 1.63. According to quantitative genetic theory (Hallauer et al., 2010), the σ_*GCA*_ exploited in the second step amounted to $\sigma_{CGA_{F5:6}}=\sqrt{{\frac{7}{8}\sigma }_{GCA\text{\_}F2}^{2}}=1.1$. The total response to selection was the sum of the responses of the first and second step.

### Characterization of Field Locations

In the recent decades, Germany has become more prone to drought events with harmful effects to agro-ecosystems. Personal communication with responsible field technicians indicated adverse field conditions in some of the environments in which the genotypes of the RRGS program were tested. Therefore, GCA_Female_-by-environment interaction effects were obtained from model (5) to estimate Euclidean distances between each pair of environments.

To further investigate the range in which the environments differed regarding physical stress, we used data from meteorological and satellite-based approaches estimating the plant available water and the condition of the regional vegetation, respectively. The German drought monitor provides data on plant available water beginning from 2015. Information for the plant available water at each location was extracted from the German drought monitor for the growing season 2018/2019 (Zink et al., 2016). In addition, the Vegetation Condition Index (VCI) was employed to quantify the severity of drought stress around the test locations. Geospatial data sets based on the MOD13Q1 images were accessed from the Application for Extracting and Exploring Analysis Ready Samples (https://lpdaacsvc.cr.usgs.gov/appeears/) by USGS. Data from MOD13Q1 images were available for the growing seasons 2011/2012, 2012/2013, and 2018/2019, qualifying them for the comparison of the HYWHEAT and RRGS environments. For each location, an area of 500 ha centered for the coordinates of the test site was selected. The VCI based on the Enhanced Vegetation Index (EVI) was obtained from the equation:


(6) $$\begin{array}{r} VCI_{i}=\frac{EVI_{i}-EVI_{\min}}{EVI_{\max}-EVI_{\min}}, \end{array}$$

where *VCI*_*i*_ referred to the VCI on day *i*, *EVI*_*i*_ referred to the EVI on day *i*, *EVI*_min_ referred to the minimum EVI in the area observed in the period 2010–2019, and *EVI*_max_ referred to the maximum EVI in the area observed in the period 2010–2019. The recommended practice for drought monitoring using the VCI was applied as suggested by the United Nations Office for Outer Space Affairs (2021). The mean value of the selected area around the test site was applied in further considerations.

Based on the data for PAW and VCI, matrices with the individual weather profile of each environment were constructed. From these matrices, principal component analyses were performed, and complete-linkage clusters based on the Euclidean distances were obtained to identify environments with special conditions.

## Results

### Analysis of Population Structure Revealed Genomic Traces of Selection

**Figure 1:**
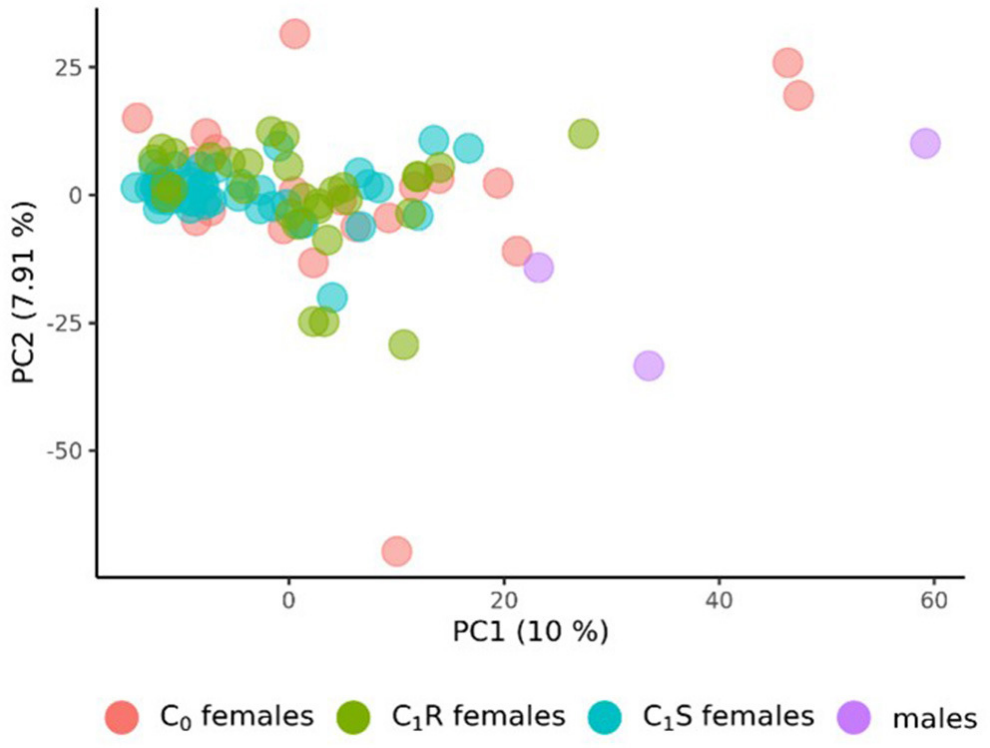
Principal Component Analysis (PCA) of the 20 founder wheat lines (C_0_ females), the 3 male lines, the 30 female lines drawn from random after recombining the 20 founder lines (C_1_R), and the 49 female lines from the first selection cycles (C_1_S). PCA were derived from the eigenvectors of the 3 male and 20 female founder lines. The proportion of variance displayed by the principal components (PC) were presented in brackets.

The population structure of the 3 male tester lines, the 20 founder female lines (C_0_) of the RRGS program, their 30 resulting randomly drawn (C_1_R) recombined, and 49 selected progenies (C_1_S) was analyzed based on 4,031 polymorphic SNP markers. The principal component analysis derived from the eigenvectors of the parental lines revealed that male and female lines tended to be separated by the first principal component (Figure 1). With respect to the second principal component, C_1_R was more widely spread than C_1_S. Overall, C_1_S appeared to be more separated from the male parents than C_1_R.

### Phenotypic Data Indicated Pronounced Interactions Between Genotypes and Environments

Genomic repeatabilities were moderate to high, ranging from 0.13 in Wohlde to 0.51 in Hadmersleben with an average of 0.34 in lines and ranging from 0.17 in Mintraching to 0.58 in Adenstedt with an average of 0.34 in hybrids (Supplementary Table 1). This underlines the overall high quality of the yield trials. Interestingly, we observed that correlations between grain yields in each environment were low for some pairs (Table 1). For example, grain yields of lines and hybrids studied at Wohlde and Hadmersleben were not significantly correlated (*r* = 0.09; *P* > 0.36 for lines; and *r* = −0.08; *P* > 0.20 for hybrids). The grain yield trial conducted at Hadmersleben was not an outlier but correlated significantly with the grain yield trial conducted at Boehnshausen (*r* = 0.51; *P* < 0.001 for the lines; and *r* = 0.23; *P* < 0.001 for the hybrids), a second location in Saxony-Anhalt. These pronounced differences among locations were also visible in the contribution of genotype-by-environment interaction effects (G × E) to the phenotypic variance (Table 2). Genotypic variances $\sigma_{G}^{2}$ were significantly greater than zero (*P* < 0.01, Table 2) for lines as well as hybrids, with $\sigma_{G}^{2}$ being 5.85-times smaller in hybrids than in lines. The ratio of $\sigma_{GxE}^{2}/\sigma_{G}^{2}$ amounted to 0.81 in lines and the ratio of $\sigma_{GCA\left(Female\right)xE}^{2}/\sigma_{GCA\;\left(Female\right)}^{2}$ to 1.13 for general combining ability effects of the females, which was of special interest during the selection. This underlines the substantial contribution of genotype-by-environment-interaction effects to the phenotypic variance. The estimated heritability (*h*^2^) was high for lines (0.84) and moderate (0.54) for hybrids.

**Table 1:** Pearson moment correlations between grain yield of 109 wheat lines (below diagonal) and 264 hybrids (above diagonal) evaluated at six locations in the year 2019 to assess the selection gain of the reciprocal recurrent genomic selection program.

**Inbred/hybrid**	**Adenstedt**	**Boehnshausen**	**Hadmersleben**	**Mintraching**	**Sossmar**	**Wohlde**
Adenstedt	1.00	−0.01	0.05	0.10	−0.02	0.17**
Boehnshausen	0.42***	1.00	0.23***	0.15*	0.12*	−0.07
Hadmersleben	0.22*	0.51***	1.00	0.13*	0.14*	−0.08
Mintraching	0.29**	0.22*	0.26**	1.00	0.14*	−0.01
Sossmar	0.54***	0.55***	0.39***	0.17”	1.00	−0.01
Wohlde	0.44***	0.12	0.09	0.32***	0.24*	1.00

”, *, **, and *** significantly different from zero at the 0.05, 0.01, 0.001, and 0.0001 level of probability.

**Table 2:** Estimates of variance components (residual variance indicated as σ_e_) and heritability (*h*2) for winter wheat for grain yield (dt/ha).

**Source**	**Grain yield**	**Grain yield**
	**(dt/ha)**	**(dt/ha)**
	**6 locations**	**4 locations**
**Lines**		
$\sigma_{\text{LINES}}^{2}$	17.21***	17.91***
$\sigma_{\text{LINESxE}}^{2}$	14.01***	10.05***
*h*^2^(Lines)	0.84	0.76
**F**_**1**_ **hybrids**		
$\sigma_{\text{SCA}}^{2}$	1.07**	1.05
$\sigma_{\text{SCAxE}}^{2}$	6.86**	7.50
$\sigma_{\text{GCA}\left(\text{Female}\right)}^{2}$	1.73**	2.14*
$\sigma_{\text{GCAxE}\left(\text{Female}\right)}^{2}$	1.97***	2.20*
$\sigma_{\text{GCA}\left(\text{Male}\right)}^{2}$	0.00	0.00
$\sigma_{\text{GCAxE}\left(\text{Male}\right)}^{2}$	1.57^NS^	1.95^NS^
$\sigma_{\text{HYBRIDS}}^{2}$	2.94	3.20
$\sigma_{\text{HYBRIDSxE}}^{2}$	10.40	11.65
$\sigma_{\text{e}}^{2}$	5.73***	5.77***
*h*^2^(hybrids)	0.54	0.44

*Parents and checks were grouped together as lines. The panel was evaluated at 6 locations and comprised 109 lines (7 checks, 99 females and 3 males) and 264 hybrids. In a further analysis, only 4 locations with no stressful growing conditions were investigated. NS, Not significant.*

*, **, and *** significantly different from zero at the 0.01, 0.001, and 0.0001 level of probability.

### Drought Stress Was Associated With the Pattern of Genotype-by-Environment Interactions

**Figure 2:**
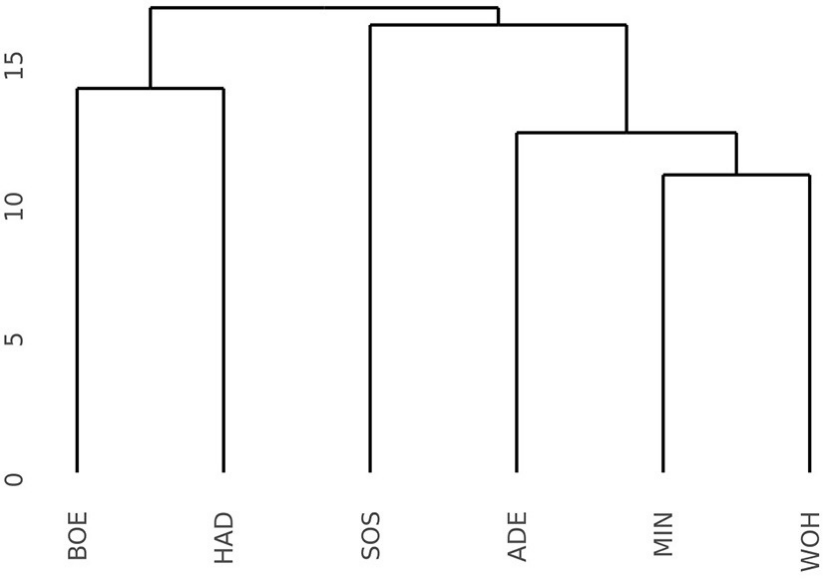
Dendrogram based on the Euclidean distances among six locations estimated using the GCA_Female_-by-environment interaction effects from the grain yield trials performed in the year 2019 to assess the selection gain of the reciprocal recurrent genomic selection program. The locations were ADE, Adenstedt; BOE, Boehnshausen; HAD, Hadmersleben; MIN, Mintraching; SOS, Sossmar; WOH, Wohlde.

**Figure 3:**
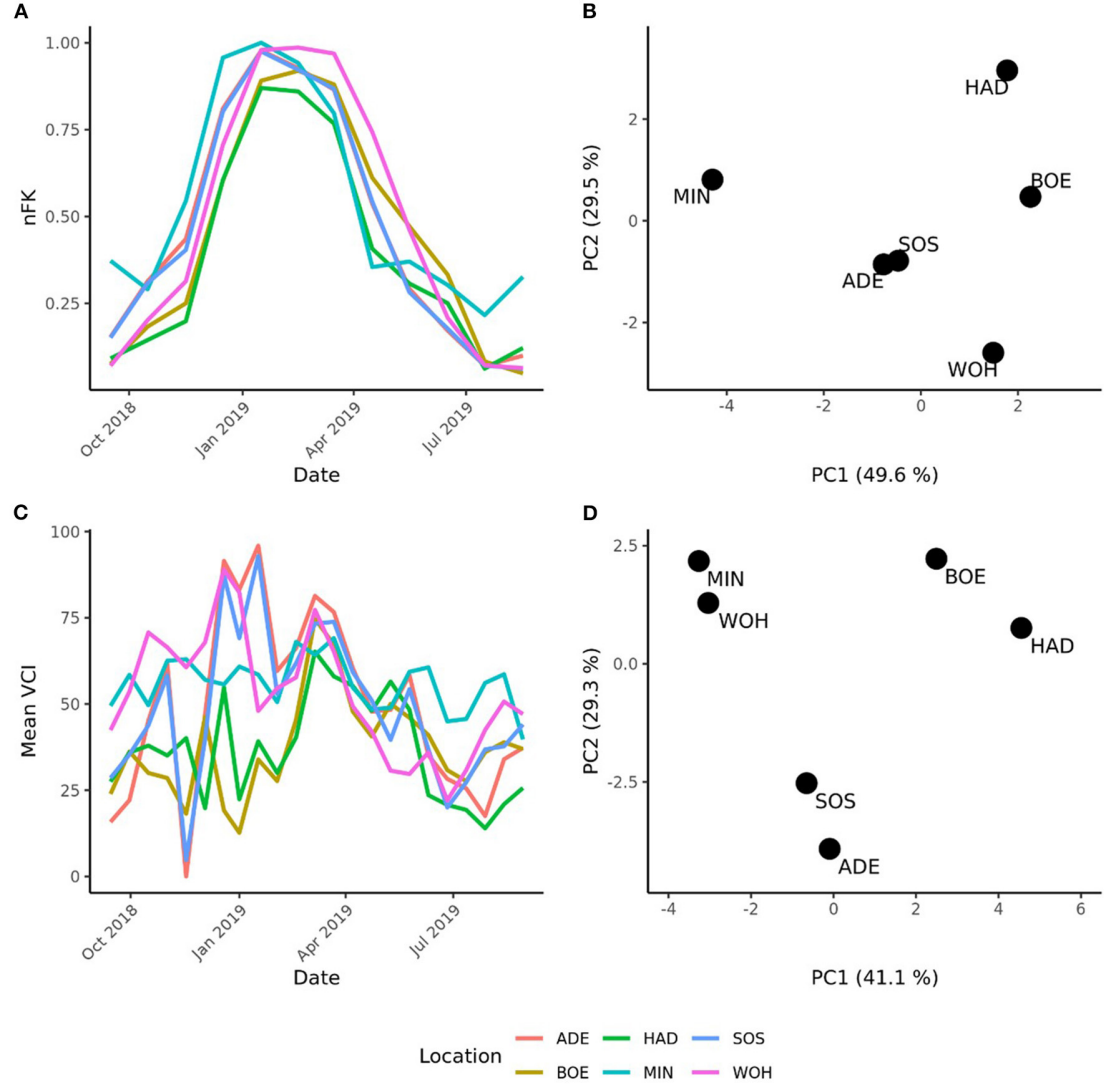
Characterization of the locations used to assess the selection gain of the reciprocal recurrent genomic selection program. **(A)** Line plot of the plant available water (PAW) in the soil and **(B)** a principal component analyses (PCA) based on the PAW profiles of the locations recorded in the growing season [September 1st in the year of sowing (2018) to September 1st in the year of harvest (2019)]. **(C)** Line plot of the mean vegetation condition index (VCI), and PCA based on the mean VCI profiles of the locations recorded in the growing season **(D)**. The locations were indicated as ADE, Adenstedt; BOE, Boehnshausen; HAD, Hadmersleben; MIN, Mintraching; SOS, Sossmar; WOH, Wohlde.

The pronounced differences among locations encouraged us to investigate the pattern of interaction effects between genotypes and environments in more detail. Due to the exploitation of additive effects in the recurrent genomic selection program, we focused on the interaction effects between the GCA effects of females with environments and performed a cluster analysis. The analysis revealed that the Boehnshausen and Hadmersleben locations formed a distinct group, separate from the other locations of the RRGS experiment (Figure 2). We assessed the clustering of the locations in more detail by analyzing two published meteorological and satellite-based parameters: the plant available water in the soil (PAW) and vegetation condition index (VCI). Boehnshausen and Hadmersleben were the locations with the lowest PAW during the early growing season (Figure 3A) and both locations also clearly clustered separately from the remaining locations when applying a principal component analyses based on the PAW of the entire growing season (Figure 3B). A similar picture was observed for the VCI profiles. Boehnshausen and Hadmersleben showed low VCI values throughout the growing season and distinguished from the other locations in particular during the autumn and winter months of the growing season (Figure 3C). The principal component analyses based on the VCI profiles of the entire growing season separated the Boehnshausen and Hadmersleben locations from the remaining ones (Figure 3D). Thus, the pronounced genotype-by-environment interactions were most likely caused by severe drought stress occurring in the region of Saxony-Anhalt in the growing season 2018/2019.

### Pattern of Genotype-by-Environment Interactions for Integrated Phenotypic Data of the Training and the RRGS Populations

The HYWHEAT training population was phenotyped at five locations in the 2011/2012 season and at six locations in the season 2012/2013, and the RRGS program was evaluated at six locations in the 2018/2019 season. Three overlapping locations albeit in different years were used for both, the HYWHEAT and for the RRGS trials. Interestingly, for the overlapping genotypes (27 for lines and 48 for hybrids) between the HYWHEAT and the RRGS experiments, we observed a much higher correlation between grain yield estimated in the growing seasons 2011/2012 and 2012/2013 within the HYWHEAT experiment (*r* = 0.49; *P* < 0.00 for lines and *r* = 0.43; *P* < 0.00 for hybrids) than between the RRGS experiment and the HYWHEAT experiment in 2011/2012 (*r* = −0.04; *P* < 0.80, for lines and *r* = 0.08; *P* < 0.80, for hybrids) and in 2012/2013 (*r* = 0.05; *P* < 0.40 for lines and *r* = −0.17; *P* < 0.80, for hybrids). A closer look at the correlations between grain yield of the RRGS experiment in each environment and the HYWHEAT experiments revealed strong interaction effects with years (Table 3). The RRGS experiment conducted in Wohlde and Adenstedt showed the highest correlations with the HYWHEAT experiments with a decreasing trend toward Mintraching, Sossmar, Boehnshausen, and Hadmersleben.

**Table 3:** Correlations of phenotypic data from single environments of the RRGS experiments (2018–2019) with phenotypic data from HYWHEAT experiments and with single years of the HYWHEAT experiment.

	**RRGS: 2018–2019**	**Hywheat: 2012**	**Hywheat: 2013**	**Hywheat: total**
Lines	Adenstedt	0.24	0.30	0.40*
	Boehnshausen	0.04	−0.14	−0.11
	Hadmersleben	0.03	−0.30	−0.24
	Mintraching	0.38	−0.07	0.11
	Sossmar	0.11	−0.11	0.03
	Wohlde	0.43*	0.41*	0.54**
Hybrids	Adenstedt	0.37*	0.37**	0.47***
	Boehnshausen	-0.26”	-0.27”	−0.32*
	Hadmersleben	−0.20	−0.23	−0.32*
	Mintraching	−0.20	−0.04	−0.13
	Sossmar	−0.09	−0.07	−0.07
	Wohlde	0.13	0.27”	0.24

*A number of 27 overlapping lines and 48 overlapping hybrids were included into the estimation.*

”, *, **, and *** significantly different from zero at the 0.05, 0.01, 0.001, and 0.0001 level of probability.

**Figure 4:**
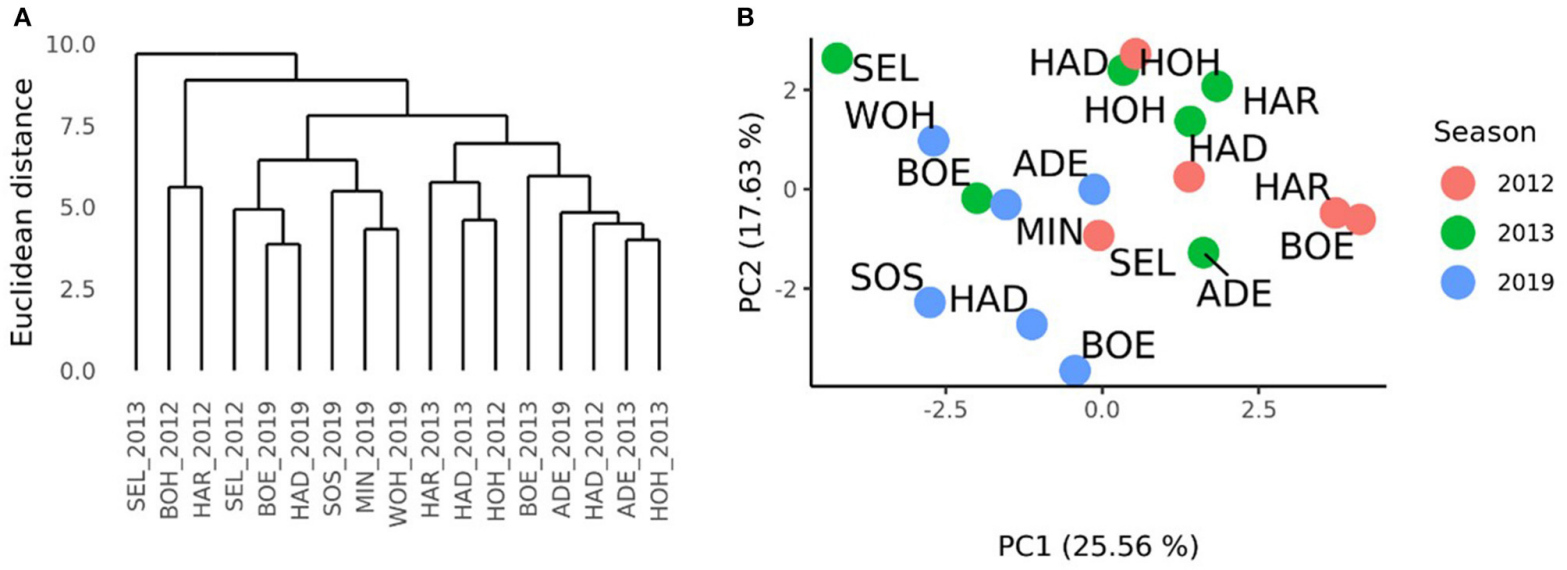
Characterization of the environments of the HYWHEAT and RRGS experiments of the growing seasons 2011/2012, 2012/2013, and 2018/2019, based on the phenotypic performances of overlapping tested hybrids. **(A)** Dendrogram based on the Euclidean distances among 17 location times year combinations (location_year) estimated using the GCA_Female_-by-environment interaction effects from the grain yield trials performed in the year 2012 and 2013 for the training population (HYWHEAT) and in the year 2019 to assess the selection gain of the reciprocal recurrent genomic selection program. **(B)** PCA based on the GCA_Female_-by-environment interaction effects of 16 location times year combinations. The locations were indicated as ADE, Adenstedt; BOE, Boehnshausen; HAD, Hadmersleben; HA*R*, Harzhof; HOH, Hohenheim; MIN, Mintraching; SEL, Seligenstadt; SOS, Sossmar; WOH, Wohlde.

A complete-linkage clustering based on the Euclidean distances estimated using the GCA_Female_-by-environment interaction effects was performed to further investigate the relationships among the environments of the HYWHEAT and the RRGS experiments (Figure 4A). The location Seligenstadt in 2013, and Boehnshausen in 2012 and Harzhof in 2012 formed outgroups. Apart from Seligenstadt in 2012, which grouped together with the environments Seligenstadt, Boehnshausen, Hadmersleben, Sossmar, Mintraching, and Wohlde from the RRGS experiment, the remaining HYWHEAT environments constituted a distinguished cluster including the environment of Adenstedt in 2019. A PCA based on the GCA_Female_-by-environment interaction effects showed that apart from Seligenstadt in 2013, the environments of the HYWHEAT experiment grouped together with the RRGS environments Adenstedt, Mintraching and Wohlde in 2019 (Figure 4B). The RRGS environments Boehnshausen, Hadmersleben and Sossmar grouped separately from the remaining environments of the RRGS and the HYWHEAT experiments.

**Figure 5:**
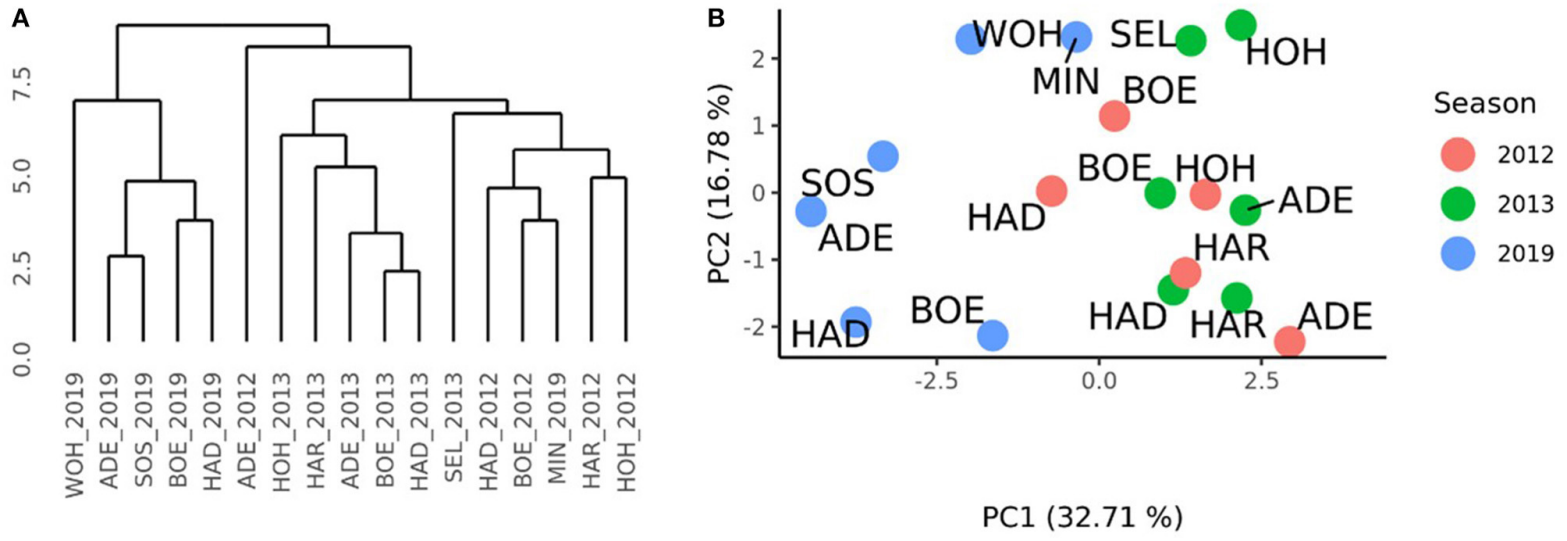
Characterization of the environments of the HYWHEAT and RRGS experiments of the growing seasons 2011/2012, 2012/2013, and 2018/2019, based on satellite-based images. **(A)** Dendrogram based on the mean vegetation condition index (VCI) profiles of 16 location times year combinations (location_year) used to perform grain yield trials in the year 2012 and 2013 for the training population and in the year 2019 to assess the selection gain of the reciprocal recurrent genomic selection program. **(B**) PCA based on the mean VCI profiles of 16 location times year combinations. The locations were indicated as ADE, Adenstedt; BOE, Boehnshausen; HAD, Hadmersleben; HA*R*, Harzhof; HOH, Hohenheim; MIN, Mintraching; SEL, Seligenstadt; SOS, Sossmar; WOH, Wohlde.

A distance matrix obtained from the VCI profiles of the 17 environments of the RRGS and the HYWHEAT experiments was calculated. The comparison to the distance matrix derived from the GCA_Female_-by-environment interaction effects revealed a correlation of 0.17 which was significantly different from zero (*P* < 0.01) according to a Mantel test (Mantel, 1967). The cluster which was derived from the VCI profiles of the 17 environments indicated the presence of two subgroups among the HYWHEAT and RRGS experiments (Figure 5A). The environments of the RRGS experiment grouped apart from the HYWHEAT experiments, with the environment of Mintraching in 2019 behaving exceptionally as it was situated within the HYWHEAT experiments. Within the HYWHEAT experiments, the location Adenstedt of the growing season 2011/2012 appeared as outgroup. The remaining HYWHEAT environments formed two subgroups distinguished mostly by the year of the evaluation. A PCA was executed based on the VCI profiles of all environments in which the genotypes were tested during the HYWHEAT and RRGS experiments (Figure 5B). This analysis exposed shifts of the growing conditions across the growing seasons in which the genotypes were evaluated. Based on the 1^st^ principal component, the environments in the RRGS experiment showed to be largely separated from all remaining environments from the HYWHEAT experiments. Only Mintraching situated closely to some of the HYWHEAT experiments. The 2^nd^ principal component separated the RRGS experiments into three groups: Mintraching and Seligenstadt, Sossmar and Adenstedt, and Boehnshausen and Hadmersleben. The first principal component explained 32.71% of the variance, the second principal component explained 16.78% of the variance.

### Selection of Test Locations Affected the Assessment of Breeding Success

Evaluation of effectiveness of RRGS was conducted at six locations during the 2018/2019 growing season, between which pronounced genotype-by-environment interaction effects were observed. Moreover, the 2018/2019 growing season locations showed high genotype-by-year interactions compared to the HYWHEAT experiments conducted in the 2011/2012 and 2012/2013 growing seasons, based on which the genomic selection model was trained. In particular, the Boehnshausen and Hadmersleben locations of the 2018/2019 growing season showed low correlations to the environments of the HYWHEAT experiment (Table 3). By comparing the BLUEs for the overlapping genotypes of the RRGS experiment with the BLUEs from the HYWHEAT experiment, correlations of 0.13 and −0.10 were observed for lines and hybrids, respectively. After excluding the locations Boehnshausen and Hadmersleben from the RRGS experiment, correlations between the RRGS experiment and the HYWHEAT experiment based on overlapping genotypes increased to 0.37 for lines and 0.21 for hybrids. Furthermore, exclusion of the Boehnshausen and Hadmersleben locations resulted in a drop of $\sigma_{GxE}^{2}/\sigma_{G}^{2}$ from 1.13 to 1.02 for the GCA of the female lines, indicating a lower proportion of genotype-by-environment interactions among the remaining locations of the RRGS experiment (Table 2). These findings encouraged us to investigate the influence of genotype-by-environment interactions on the selection gain of the RRGS breeding programs. To this end, we estimated the selection gain based on phenotypic data collected in all six environments of the RRGS experiment and alternatively we excluded two environments with negative average correlations to the single environments of the HYWHEAT data set and estimated the selection gain based on the remaining four locations.

**Figure 6:**
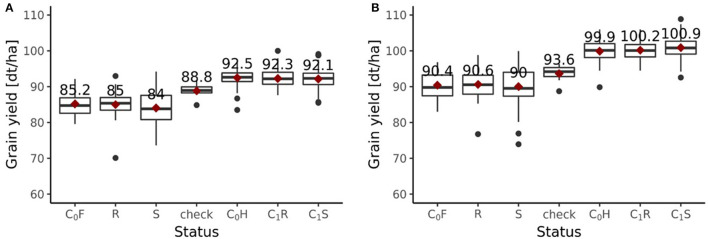
Grain yield performance depending on the status of genotypes evaluated in the 2019 experiment. **(A)** Performances of the fractions from the breeding population with all six environments of 2018/2019 included. **(B)** Performances of the fractions from the breeding population with only 4 environments of 2018/2019 included. Status indicates the affiliation of each group of genotypes to a specific fraction within the breeding program. Female parent lines from the C_0_ cycle are indicated as C_0_F, female parent lines from the randomly selected fraction of the C_1_ cycle are indicated as R, female parent lines from the genomic-selected fraction of the C_1_ cycle are indicated as S, checks are indicated as “check,” hybrids from the C_0_ cycle are indicated as C_0_H, hybrids from the randomly selected fraction of the C_1_ cycle are indicated as C_1_R, hybrids from the genomic-selected fraction of the C_1_ cycle are indicated as C_1_S.

**Figure 7:**
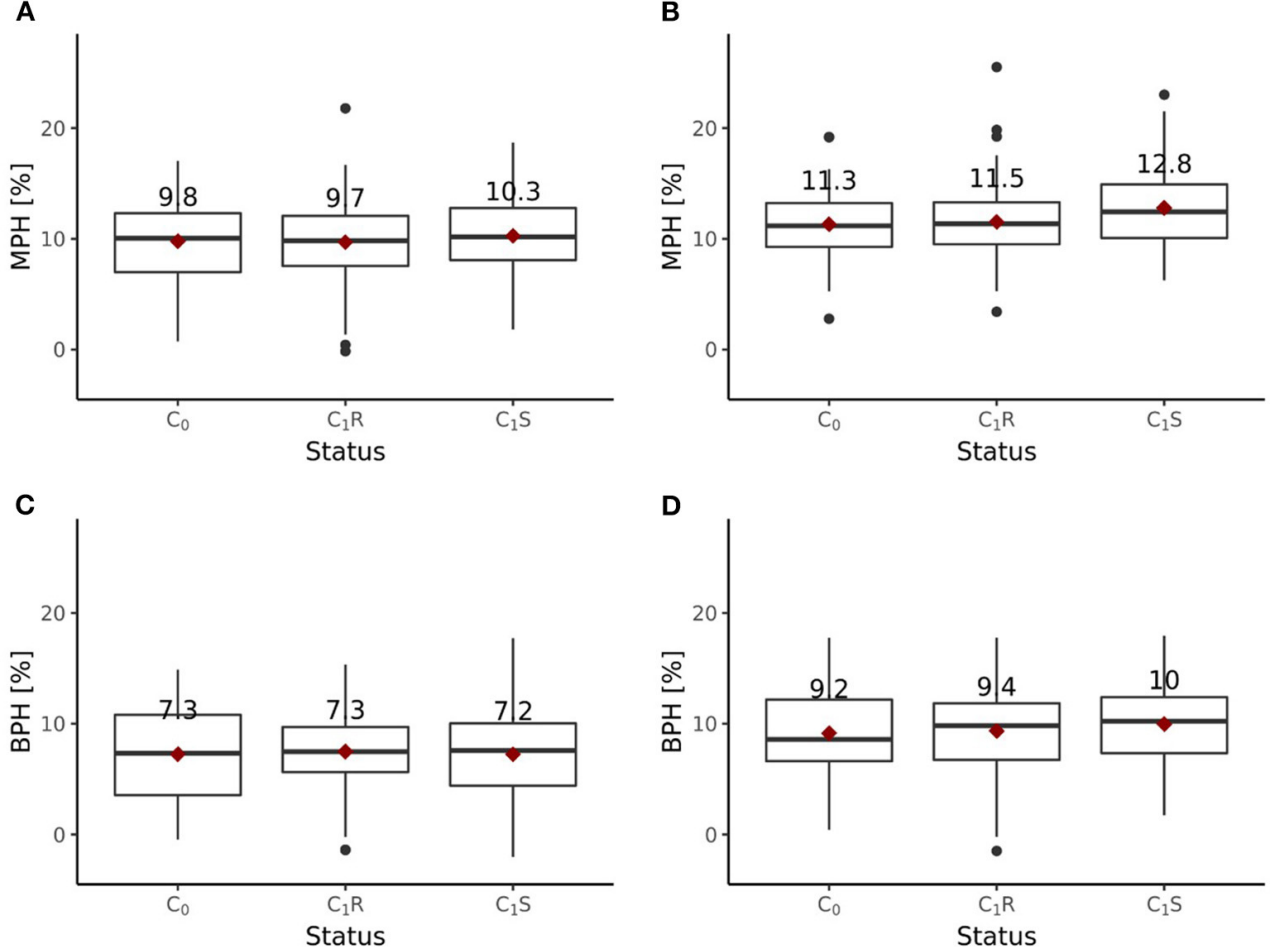
Midparent heterosis (MPH) and better parent heterosis (BPH) for hybrids generated in the reciprocal recurrent selection program. MPH [%] estimated based on trials performed **(A)** at 6 locations and **(B)** 4 locations, excluding 2 stress environments. BPH [%] estimated based on trials performed **(C)** at 6 locations and **(D)** 4 locations, excluding 2 stress environments.

Including all six environments from the growing season 2018/2019, the randomly drawn female lines of the C_1_ cycle showed comparable (*P* > 0.1) average yields as the female parent lines of the C_0_ cycle (Figure 6A). The genomically selected females showed no significant differences of 1.0 dt ha^−1^ (*P* > 0.1) average yields compared to the randomly selected female lines. Surprisingly, genomically selected female lines of the C_1_ cycle showed lower (*P* > 0.1) average yields than the female lines of the C_0_ cycle. Both differed by 1.15 dt ha^−1^. The average yield of the C_0_-hybrids, the genomic-selected fraction of the C_1_-hybrids (C_1_S) and the randomly drawn fraction of the C_1_-hybrids (C_1_R) did not show any significant (*P* > 0.1) difference. The midparent heterosis was not significantly (*P* > 0.1) larger for C_1_S (10.3%) as compared to C_1_R (9.7%) and C_0_-hybrids (9.8%) (Figure 7A). The same was observed for better parent heterosis (Figure 7C).

Excluding the two outlier locations from the growing season 2018/2019, randomly drawn female lines of the C_1_ cycle showed comparable (*P* > 0.1) average yields as the female parent lines of the C_0_ cycle (Figure 6B). Genomically selected female lines of the C_1_ cycle and randomly selected female lines of the C_1_ cycle showed no significantly different (*P* > 0.1) grain yield performance. The female parent lines of the C_1_ cycle performed comparable (*P* > 0.1) to the female parent lines of the C_0_ cycle. While C_1_R hybrids showed no significant difference (*P* > 0.1) in average yield performance compared to C_0_ hybrids, C_1_S hybrids outperformed (*P* < 0.05) C_0_ hybrids by 1.0 dt ha^−1^, achieving a selection gain of 1%. Moreover, C_1_S hybrids outperformed (*P* < 0.1) C_1_R hybrids by 0.7 dt ha^−1^. Midparent heterosis was not significantly different (*P* > 0.1) in C_1_R (11.5%) compared to C_0_ (11.3%), while C_1_S (12.8%) showed a clear advancement and performed significantly better than C_0_ (*P* < 0.05) and C_1_R (*P* < 0.05) (Figure 7B). A different pattern was observed for better parent heterosis. C_0_ (11.3%) and C_1_R performed comparable (*P* > 0.1). C_1_S (10.0%) did not perform significantly different from C_0_ (*P* > 0.1) and C_1_R (*P* > 0.1) (Figure 7D).

The observed selection differential and hence the observed response to selection varied depending on which environments were considered for the evaluation. When all six environments were included, it amounted to $R_{obs\text{\_}6E}=-0.4\;dt\;ha^{-1}$. When environments with severe stress conditions were excluded and only four environments were considered, the observed selection differential and hence observed response to selection was $R_{obs\text{\_}4E}=1.0\;dt\;ha^{-1}$.

## Discussion

We conducted one cycle of an RRGS program in wheat, including field evaluation of the resulting hybrids, which took a total of 6 years from the first crosses. It is important to note that each subsequent selection cycle lasts only one additional year at most, which illustrates the great opportunity to accelerate classical RRS programs. The RRGS program focused exclusively on the female pool and can be viewed as a special case of RRGS in which only the allele frequencies in the pool of female parent lines have been shifted with respect to the frequencies of favorable alleles in the pool of male parent lines.

This situation implies consequences for the determination of selection directions, especially in the case of overdominance, *k* > 1, with $k=\frac{d}{a}$, where *d* denotes the dominance effect and *a* denotes the additive effect. If overdominance is present at a given locus, RRGS aims to fix different alleles in the pool of female parental lines and in the pool of male parental lines, thus guarantees the desired complementarity among the two heterotic groups. For loci with *k* > 1, at which the pool of male parent lines has a fixed allele, RRGS will result in the fixation of the complementary allele in the pool of female parent lines. If the allele is not fixed in the pool of the male lines, and no selection is applied to the pool of male parental lines, complementarity among the heterotic groups cannot be achieved.

If 0 < *k* ≤ 1, i.e., in the presence of partial dominance, RRGS aims to ultimately fix the favorable allele in both heterotic groups. In the case where the male heterotic group is not fixed for the favorable allele, the optimal configuration cannot be achieved if the male heterotic group is not subject to selection.

For loci that exhibit negative dominance, i.e., *k* < 0, the desired selection direction is to fix the favorable allele in both heterotic groups. Complications arise when the unfavorable allele is present in the male heterotic group. Furthermore, if *k* < −1, i.e., negative overdominance is present, RRGS is directed toward fixation of the favorable allele only if the frequency, *p*, of the favorable allele is above the threshold *p* > (*k* + 1)/2*k* (Rembe et al., 2019).

In the present breeding program, the male heterotic group was kept constant between the C_0_ and the C_1_ cycle. As described above, this approach would not be expedient to reach the ideal allelic configurations between the two heterotic groups. However, the applied selection scheme is capable to evaluate the effectiveness of a selection that is conducted with respect to the allele frequencies within both heterotic groups. Therefore, the experimental design can serve as a model case for an RRGS breeding program.

The results of the field trials indicate that heterosis increased through RRGS (Figure 7). The selected fraction of the C_1_S hybrids showed significantly higher midparent heterosis than the C_0_ hybrids, but no significantly different better parent heterosis. In contrast, the C_1_R hybrids did not show increased midparent or better parent heterosis compared to the C_0_ hybrids. These findings highlight that the implemented selection models, which focused on additive and dominance effects, had an impact.

To evaluate the success of the RRGS program in more detail, the expected response to selection was compared to the observed response to selection. The expected response considering genomic selection at the F_2_ and F_5:6_ levels was $R_{\exp}=2.6\;dt\;ha^{-1}$, which was much lower than the observed response considering all six environments ($R_{obs\text{\_}6E}=-0.4\;dt\;ha^{-1}$) or the four environments ($R_{obs\text{\_}4E}=1.0\;dt\;ha^{-1}$). The difference between *R*_*obs*_6*E*_ and *R*_*obs*_*E*_ clearly suggests that different growing conditions in the environments impacted the assessment of the response to selection. But even *R*_*obs*_4*E*_ was 2.6 times smaller than the expected response of selection *R*_*exp*_, indicating that the implemented RRGS breeding program falls short of expectations. This observation can be mainly attributed to a high amount of genotype-by-year interactions between the 2011/2012, 2012/2013, and 2018/2019 experiments as highlighted in the detailed analyses of the interaction between genotypes and years (Figures 4, 5). Multi-year testing could be an option to reduce the risk of unsuitable selection decisions.

So far, there are no experimental studies that have evaluated the effectiveness of an RRGS breeding program in cereals. In an RGS breeding program in wheat for the less complex trait grain fructans compared to grain yield, significant genotype-by-environment interactions were observed with little effects on prediction accuracies (Veenstra et al., 2020). In contrast, in an RRS program in tropical maize focusing on grain yield, Kolawole et al. (2018) also observed that genotype-by-environment interactions negatively affected the observed response to selection.

As an alternative approach to estimate the expected response of selection, realized prediction ability was examined as the correlation between predicted average hybrid performances and the observed average hybrid performance of the 30 randomly drawn female parent lines from the C_1_ cycle. When all six environments of the season 2018/2019 were included in the analysis, a realized prediction ability of 0.13 was observed. Excluding environments with stressful growing conditions for the 2018/2019 data set resulted in a realized prediction ability of 0.27. These realized prediction abilities of the 2018/2019 growing season are substantially lower than the prediction abilities estimated by cross validations based on the data of the HYWHEAT experiment conducted in the 2011/2012 and 2012/2013 growing seasons (Zhao et al., 2015). This can only partly be explained by the small sample size of 30 randomly drawn female parent lines from the C_1_ cycle used to estimate the prediction abilities. Moreover, it is unlikely that the low realized prediction abilities have been caused through recombination. More likely, the lower realized prediction abilities are due to interaction effects between genotypes, locations, and years.

When the prediction abilities estimated based on the 30 randomly drawn female parent lines from the C_1_ cycle are used to estimate the expected response to selection, the value decreases to $R_{exp\text{\_}6E}=0.09\;dt\;ha^{-1}$ and $R_{exp\text{\_}4E}=1.22\;dt\;ha^{-1}$, depending on whether stressful environments are included or not. In this case, *R*_*obs*_4*E*_ was only 1.22 times smaller than the expected response of selection *R*_exp_. Consequently, it is pivotal to obtain genome-wide prediction models that are not biased due to interaction effects between genotypes, locations, and years. One promising approach to achieve this, is to account for interaction effects between genotypes and environments by implementing environmental cofactors into genome-wide prediction models (de los Campos et al., 2020). This facilitates to reduce the adverse effects due to interactions between genotypes and environments and to develop more sustainable genome-wide prediction models. In addition, aggregation of available medium size genomic and phenotypic data across different projects and perhaps even breeding programs into large data sets can help substantially to reduce confounding effects of genotype-environment interactions (Zhao et al., 2021). These adjustments seem urgently needed to further leverage the potential of RRGS.

## Data Availability Statement

The datasets presented in this study can be found in online repositories. The names of the repository/repositories and accession number(s) can be found at: https://doi.org/10.1093/database/baw033.

## Author Contributions

JR, EE, EK, CL, PT, and YZ conceived and designed the study. EE, VK, JS, PB, PV, NPh, NH, SK, NPf, and MG acquired and contributed data. MR processed the data, performed the analyses, and analyzed the results. YZ supervised the data analyses. MR, JR, and YZ interpreted the results and wrote the manuscript. EE, PT, VK, JS, PB, PV, EK, NPh, SK, NPf, CL, NH, and MG provided input. All authors contributed to the article and approved the submitted version.

## Funding

This work was funded by BMEL through BLE within the ZUCHTWERT project (Grant ID: 2814604113) and the HYFLOR project (Grant ID: 2818401A18).

## Conflict of Interest

EE and NPf were employed by KWS LOCHOW GmbH. PT, VK, and SK were employed by KWS Saat SE & Co. KGaA. JS, PB, and PV were employed by Limagrain GmbH. EK and NPh was employed by Syngenta Seeds GmbH. NH was employed by Nordsaat Saatzucht GmbH. MG was employed by Nordsaat Saatzucht GmbH and is presently employed by Strube Research GmbH & Co. KG. The remaining authors declare that the research was conducted in the absence of any commercial or financial relationships that could be construed as a potential conflict of interest.

## Publisher's Note

All claims expressed in this article are solely those of the authors and do not necessarily represent those of their affiliated organizations, or those of the publisher, the editors and the reviewers. Any product that may be evaluated in this article, or claim that may be made by its manufacturer, is not guaranteed or endorsed by the publisher.
