# Supplementary material for: Reciprocal Recurrent Genomic Selection Is Impacted by Genotype-by-Environment Interactions
Source: Front Plant Sci. 2021 Sep 24;12:703419. doi: 10.3389/fpls.2021.703419 (PMC8498042; doi:10.3389/fpls.2021.703419)
Supplement: Supplementary file 1 [file Table_1.DOCX]

**Supplementary Table 1**. Location, geographic positions and genomic repeatabilities of the RRGS experiments.

| Location | Geographic position | Genomic repeatability: lines | Genomic repeatability: hybrids |
| --- | --- | --- | --- |
| Adenstedt | latitude 52.20 N, longitude 10.18 E | 0.29 | 0.58 |
| Boehnshausen | latitude 51.85 N, longitude 10.95 | 0.35 | 0.25 |
| Hadmersleben | latitude 51.98 N, longitude 11.30 E | 0.51 | 0.47 |
| Mintraching | latitude 48.95 N, longitude 12.25 E | 0.29 | 0.17 |
| Sossmar | latitude 52.20 N, longitude 10.08 E | 0.44 | 0.27 |
| Wohlde | latitude 52.80 N, longitude 9.98 E | 0.13 | 0.31 |
